# Metagenomic Survey for Viruses in Western Arctic Caribou, Alaska, through Iterative Assembly of Taxonomic Units

**DOI:** 10.1371/journal.pone.0105227

**Published:** 2014-08-20

**Authors:** Anita C. Schürch, Debby Schipper, Maarten A. Bijl, Jim Dau, Kimberlee B. Beckmen, Claudia M. E. Schapendonk, V. Stalin Raj, Albert D. M. E. Osterhaus, Bart L. Haagmans, Morten Tryland, Saskia L. Smits

**Affiliations:** 1 Department of Viroscience, Erasmus Medical Center, Rotterdam, The Netherlands; 2 Viroclinics Biosciences, Rotterdam, The Netherlands; 3 Alaska Department of Fish and Game, Kotzebue, Alaska, United States of America; 4 Alaska Department of Fish and Game, Division of Wildlife Conservation, Fairbanks, Alaska, United States of America; 5 Research Group for Arctic Infection Biology, Department of Arctic and Marine Biology, UiT - the Arctic University of Norway, Tromsø, Norway; 6 Genøk - Centre for Biosafety, Tromsø, Norway; The Pirbright Institute, United Kingdom

## Abstract

Pathogen surveillance in animals does not provide a sufficient level of vigilance because it is generally confined to surveillance of pathogens with known economic impact in domestic animals and practically nonexistent in wildlife species. As most (re-)emerging viral infections originate from animal sources, it is important to obtain insight into viral pathogens present in the wildlife reservoir from a public health perspective. When monitoring living, free-ranging wildlife for viruses, sample collection can be challenging and availability of nucleic acids isolated from samples is often limited. The development of viral metagenomics platforms allows a more comprehensive inventory of viruses present in wildlife. We report a metagenomic viral survey of the Western Arctic herd of barren ground caribou (*Rangifer tarandus granti*) in Alaska, USA. The presence of mammalian viruses in eye and nose swabs of 39 free-ranging caribou was investigated by random amplification combined with a metagenomic analysis approach that applied exhaustive iterative assembly of sequencing results to define taxonomic units of each metagenome. Through homology search methods we identified the presence of several mammalian viruses, including different papillomaviruses, a novel parvovirus, polyomavirus, and a virus that potentially represents a member of a novel genus in the family *Coronaviridae*.

## Introduction

Barren-ground caribou (*Rangifer tarandus granti*) is a wild-living subspecies of reindeer in Alaska and Canada. The Western Arctic caribou herd (WACH) in Alaska is the largest caribou herd in North America, occupying the northwestern part of the state. The size of the WACH reached a population apex of ∼490,000 individuals in 2003, but has declined to ∼325,000 animals in 2011, which was likely due to varying climate conditions, population density, predation, and disease outbreaks, or a combination of these factors. The WACH follows general movement patterns for each season of the year ranging over a 140,000 square mile area bounded by the Arctic Ocean, lower Yukon River, and the trans-Alaska pipeline. In spring, mature cows travel north to the calving grounds, and bulls and immature cows move north-west to the summer range. After calving, cows and calves travel west to join the remainder of the herd. By fall the herd is most dispersed and starts migrating southwest to the winter grounds. The caribou are an integral part of the ecology and sociology of the circumpolar North and since 2000, an estimated 10,000–15,000 Western Arctic caribou have been killed each year for subsistence within the range of the herd (Alaska state department). Caribou represent a wildlife reservoir for viruses that can be transmitted to semi-domesticated reindeer (*R. t. tarandus*), and in some cases possibly also to other wild ruminants [1]. Since 1996, the WACH expanded their seasonal migratory routes, embracing the eastern half of Seward Peninsula and westwards, where caribou had been absent for more than 150 years [2]. This has increased the contact with herded reindeer, which to a large extent have out-migrated with the caribou. Both the caribou and the reindeer herds are free-ranging. Interaction and interbreeding between wild and semi-domesticated reindeer species is common and has also occurred in Alaska (Hunderdtmark and Groves, unpublished data).

Surveillance of viruses in caribou is scarce [3], mostly based on serological assays identifying antibodies against circulating viruses or, possibly, cross-reacting agents. This baseline knowledge is relevant, especially if diseased animals are identified or if epizootics appear. Knowledge on virus infections in caribou, and especially the WACH with its contact to reindeer herding on the Seward Peninsula, is crucial, because diseases may play a role in the declining population, and also because some viruses are expected to be transmitted between reindeer/caribou and other ruminant species. Few studies have been conducted to try to identify viruses in caribou through isolation, polymerase chain reaction (PCR), or metagenomic surveys [4]. Random amplification of nucleic acids and next-generation sequencing with metagenomics analyses has been successful in recent years in identifying new viruses from wild animals [5]–[7]. However, the availability and volume of samples obtained from living wild animals is often limited and viral loads in samples may be low, allowing only few virus characterization steps beyond the metagenomic assay. By optimally assembling sequences in metagenomic studies, the likelihood of identifying and characterizing viral genomes could potentially be increased.

In contrast to whole genome sequencing, metagenomes comprise a variety of differentially abundant species, and there can be substantial interpopulation diversity within a single species. Whether two reads originate from the same gene as an entity is depending on many factors; abundance of the organism in the sample, size and copy number of the gene in the original sample [8], [9], effectiveness of enrichment strategies [10], amplification biases introduced during random amplification [11]–[13], biases inherent to next-generation sequencing protocols [14], and depth of sequencing and read lengths [15]. In theory, two reads of the same taxonomic unit should be assembled into a single contig if they have sufficient overlap. Most assemblers are designed for single genome assemblies and therefore identify highly covered regions as possible repeats rather than highly abundant organisms within a metagenomic sample [16] or regions with a highly uneven coverage across the genome [12]. In addition, sequence diversity as present in a population of pathogens might hamper successful assembly, and some reports recommend that metagenomic datasets should not be assembled at all [17].

The fewer sequences subjected to *de novo* assembly, the fewer pairwise comparisons have to be made, thereby reducing time of computation and memory usage and increasing the probability of correct assembly. Especially greedy assembly algorithms that join individual reads together, starting with the best overlapping pair, as implemented in Phrap (http://www.phrap.org) or CAP3 [18] might miss a non-optimal overlap. In contrast, overlap-layout-consensus (OLC) assemblers start with a global analysis by constructing an overlap graph from the pairwise comparisons before consensus computation [19]. However, practical implementations of OLC, like Newbler [20], Celera assembler [21] or Arachne [22], assemble contigs rather conservative, meaning that only contigs with a high fidelity are formed [19].

In this study, we applied different assembly algorithms on trimmed sequencing reads of metagenomes obtained from eye and nose swabs from caribou from the WACH, having a basically unknown health status in terms of viral infections, to determine an optimal assembly approach. An initial assembly with an overlap-layout-consensus (OLC) assembler (Newbler) followed by iterative rounds of elongation of these contigs with a well-established greedy assembly algorithm (CAP3) appeared to perform best. After convergence of the assemblies, the resulting contigs and singletons were subjected to homology searches. This approach identified a variety of mammalian viruses in eye and nose swabs of caribou, including novel viruses.

## Materials and Methods

### Sample collection and preparation

**Figure 1:**
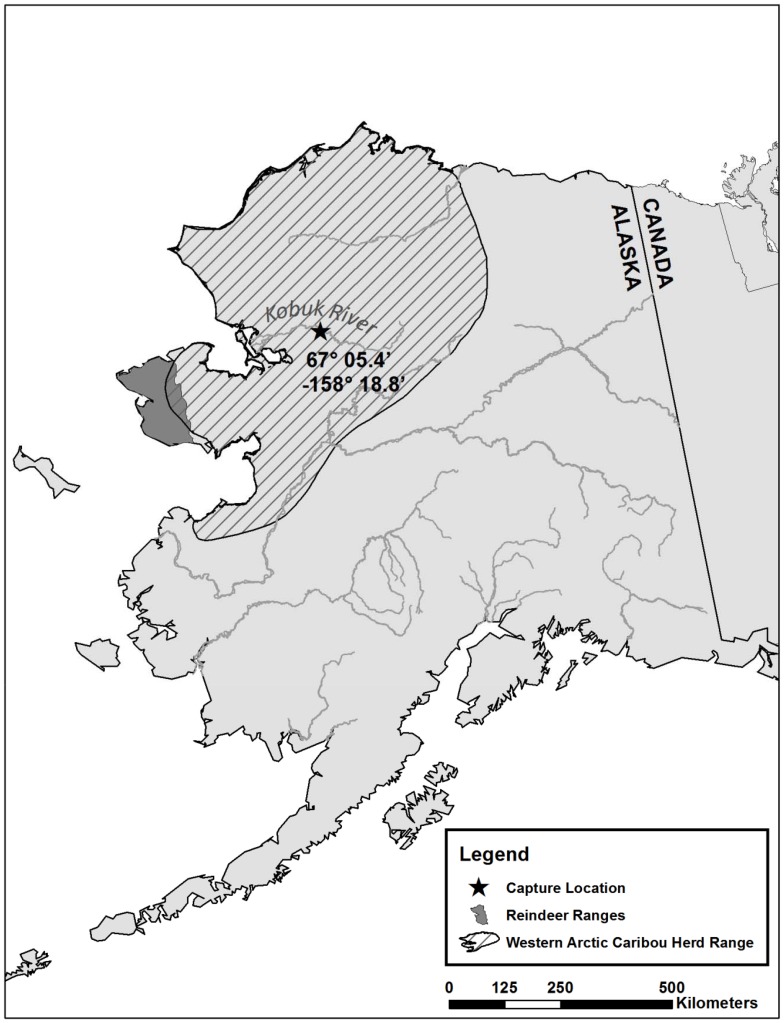
Map of the caribou and reindeer ranges, which illustrates the contact between these animals, as well as the sampling site at the Kobuk River bank.

Samples were collected from live adult caribou (n = 39, 25 females, 14 males) from the Western Arctic caribou herd. Animals were caught and held against the side of the boat when swimming across to the southern river bank of Kobuk River, at Onion Portage (67° 5.4′; -158° 18.8′), in September 2012 (Figure 1; Table S1). No specific clinic disease signs were recorded for any of the animals and the animals were assumed to be healthy. A swab sample (sterile cotton; SelfaTrade, Spånga, Sweden) was obtained from the mucosa of the lower eye lid and from the nose, 3–4 cm inside one nostril, from each animal. The swabs were transferred to sterile cryotubes containing 800 µl of cell culture medium (Eagles Minimal Essential Medium; EMEM, Biochrom, Berlin, Germany) containing antibiotics (penicillin 100 U/ml, streptomycin 100 µg/ml, gentamicin 50 µg/ml, amphotericin B 2.5 µg/ml). The swabs were kept cool at the sampling site, frozen the same day on liquid nitrogen and kept at −80°C.

### Ethics statement

In compliance with relevant laws and institutional guidelines, this project protocol was approved by the Division of Wildlife Conservation Institutional Animal Care and Use Committee (DWC ACUC 2012-031). All efforts were made to minimize animal suffering.

### Metagenome sequencing

Depletion of host nucleic acids, isolation of viral nucleic acids, sequence-independent amplification and next-generation of sequencing with a 454GS Junior (Roche) was carried out as previously described [6], [23], [24].

### Assembly

Iterative exhaustive assembly of sequences is part of a virus discovery pipeline written in the python programming language (Python 2.7) that includes trimming of reads and initial assembly with Newbler (454GS Assembler version 2.7, Roche), with standard parameters. Trimmed reads and initial contigs were subjected to assembly by CAP3 (VersionDate: 12/21/07) with standard parameters. The resulting singletons and contigs were iteratively assembled by CAP3 until no new contigs were formed. Subsequently, the trimmed reads were mapped back to the identified taxonomic units with Newbler (454 GSMapper version 2.7, Roche) using a minimum length of 75 nucleotide and otherwise standard parameters. Resulting contigs and singletons were filtered with Dustmasker which is part of the NCBI-BLAST+ 2.2.25 suite of tools for sequences that contain more than 60% low complexity sequences.

A number of 15 000 (once), 10 000 (six times) and 5000 reads (six times) were simulated with Metasim version 0.9.1 [25] from a database containing 2314 human genes, 12 microbial genomes, and 50 viral genomes, with varying copy numbers (1–50). The reads were simulated with a 454-specific error profile and a mean length of 252 bases. From these thirteen simulated metagenomes, a single read per gene or genome was extracted, resulting in datasets with between 1218 to 3288 reads, called taxonomic units. These thirteen sets, along with the 78 reindeer metagenomes, were subjected to *de novo* assembly with six different assembly strategies. “Iterative assembly” and “Iterative assembly + mapping” were performed as described above, but the former omitted the final step of mapping of the trimmed reads. The “Newbler” method was carried out with GS Assembler version 2.7, Roche, with standard parameters. For “CAP3”, sequences were assembled once with CAP3 (VersionDate 12/21/07) with standard parameters. For “CLC”, sequences were subjected to CLC Genomics Workbench Version 6.0.4 and run with an automatic word and bubble size and a minimum contig length of 75. “Mira” was performed with Mira Version 3.4.1 [26] and run with –job = denovo,genome,accurate,454 -notraceinfo.

### Metagenome analysis

After filtering of low complexity sequences, the remaining taxonomic units were subjected to a BLASTN search against a database that contained only nucleotide sequences from birds (*aves*, taxonomic ID 8782), carnivores (*carnivora*, taxID 33554), primates (*primates*, taxID 9443), rodents (*rodentia*, taxID 9989) and ruminants (*ruminantia*, taxID 9845) with an e-value cut-off of 0.001 for subtraction of potential host sequences. Sequences without hits in the host-BLAST were then subjected to a BLASTN search against the entire nt database downloaded on 07 November 2013 with an e-value cut-off of 0.001. Due to limited capacity, all sequences without hits were then subjected to a BLASTX search against viral protein sequences present in the nr database at 07 November 2013. BLAST hits were categorized by assigning a taxonomic category. Sequences with a best-BLAST hit to virus families likely to infect eukaryotes were compared to the sequence of the respective virus on nucleotide and protein level. Taxonomic units of all reindeer samples were subjected to a tBLASTX with stringent e-value cut-offs against sequences of the subfamily *Torovirinae.* Alignments were checked visually.

### Phylogenetic analysis

Alignments were prepared with MAFFT version 7 (http://mafft.cbrc.jp/alignment/server/) using default parameters (Blosum62). PhyML trees were generated using Seaview 4 software with the approximate likelihood ratio test based on a Shimodaira-Hasegawa-like procedure which used general time reversible (nt) or LG (aa) as substitution model. Nearest Neighbour interchange, subtree pruning, and regrafting-based tree search algorithms were used to estimate tree topologies, as described previously [27] with corresponding sequences of representative members of the respective virus families (Table S2). The *Nidovirus* phylogenetic Maximum Likelihood tree was created with WAG with Freqs. and Gamma distributed with Invariant sites (G+I) parameters. A collection of L1 gene sequences of a variety of papillomaviruses (AaPV1, *Alces alces* Papillomavirus 1; BPV, bovine Papillomavirus; RtPV, *Rangifer tarandus* papillomavirus; AsPV1, *Apodemus sylvaticus* Papillomavirus 1; OvPV1, *Odocoileus virginianus* Papillomavirus 1; OaPV, *Ovis aries* Papillomavirus; EcPV3, *Equus caballus* Papillomavirus 3; EePV1, *Erinaceus europaeus* Papillomavirus 1) was used for comparison. Taxonomic units with a best-BLAST hit against a member of the *Papillomaviridae* family were subjected to a BLASTN search against a collection of papillomavirus L1 gene sequences retrieved from the Papillomavirus Episteme (http://pave.niaid.nih.gov; accessed November 20, 2013).

### Nucleotide sequence accession numbers

The L1 nucleotide sequence of RtPV4, VP2 and NS1 sequences of caribou parvovirus, partial helicase sequence of caribou nidovirus, and the large T antigen sequence of caribou polyomavirus were deposited in the GenBank sequence database under accession numbers KJ719447-451, respectively.

## Results

### Comparison of different assembly algorithms

**Figure 2:**
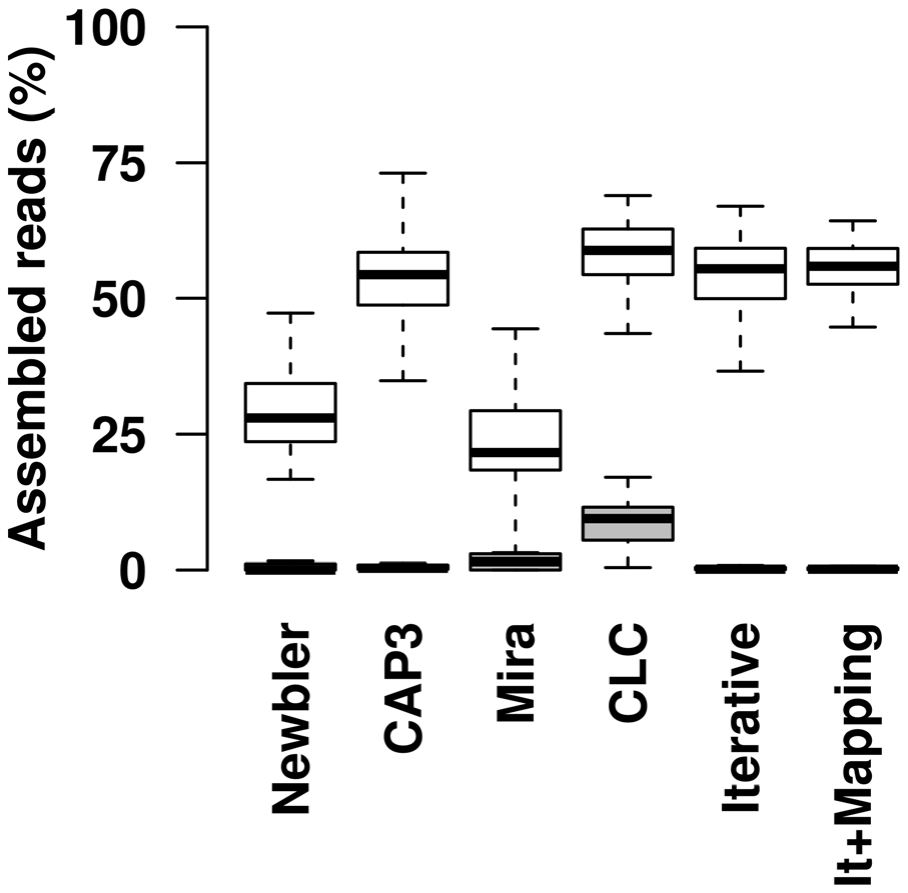
Assembly efficiency of different alignments strategies. “Newbler”, “CAP3”, “Mira”, “CLC” indicate single rounds of *de novo* assembly with the respective assembler as described in Material and Methods. “Iterative” and “It+Mapped” indicates the assembly strategy applied in this study, without and with mapping of the trimmed reads to the taxonomic units after convergence of the assembly. White: Box plots of the percentage of assembled reads based on swab mucosa samples (nose and eye) of 78 caribou from Alaska, USA. Grey: Box plots of the percentage of assembled reads from 13 simulated metagenomic dataset containing non-overlapping reads (negative controls). Top and bottom of the box indicate the first and third quantile, the band inside the box marks the median. Whiskers extend to the upper and lower quartiles, and outliers are plotted as individual points.

Sequence-independent amplification and next-generation sequencing [21], [22] was applied to both eye and nose swabs. To obtain insight in the performance of different assembly strategies, the percentage of reads that was assembled into contigs (as opposed to “singletons”) was compared for each of the samples with different assembly algorithms (Figure 2). Between 664 and 43 702 trimmed reads per sample were obtained from the caribou nose and eye swabs (Table S1). All reads were trimmed with Newbler and subjected to either a single *de novo* assembly with Newbler, CAP3, Mira, or a de Bruijn graph algorithm (CLC Genomic workbench assembler). The fifth method was composed of an initial assembly with Newbler, after which contigs and singletons were pooled and subjected to exhaustive iterative assembly with CAP3 until convergence of the assembly (“Iterative”) (Figure 2 and Figure S1). Finally, exhaustive iterative assembly was combined with mapping of the trimmed reads with GSMapper to the contigs and singletons that were the result of the converged iterative assembly (Figure 2 and Figure S1). While the exhaustive iterative assembly approach with mapping had significantly more reads assembled than Newbler alone (Mann-Whitney test, one-tailed, paired, p = 2.512 e-14) and Mira (p = 1.256 e-14), this is not the case for assembly with CLC Genomics Workbench, CAP3, or for exhaustive iterative assembly without mapping (Figure 2). Thus, the exhaustive iterative assembly approach with or without mapping and CLC Genomics Workbench seems to give the best results in terms of compression of the output for homology searches. In other words, the amount of taxonomic units that needed analyses through homology searches was reduced significantly (Figure 2).

To receive an indication for the stringency of the different assembly strategies, we simulated thirteen independent metagenomic datasets containing human, bacterial and viral sequences and extracted a single read per gene or genome. In such a dataset, by definition, any assembly would be chimeric. We conducted *de novo assembly* with the six different assembly strategies on all thirteen datasets as described above (Figure 2, grey boxes). Four assembly strategies had very low chimera rates with a median of less than 0.5% (Newbler, CAP3, iterative assembly and iterative assembly with mapping). Mira showed a median chimera rate of 1.5% and CLC Genomics Workbench median of 9.4% with the tested parameters. Thus, the exhaustive iterative assembly approach with or without mapping seemed to give the best results in terms of reliability of the assembly and compression of the output. To achieve the optimal assembly confidence level, the caribou dataset analysis was performed with exhaustive iterative assembly approach with mapping of original trimmed reads.

### Viral content of metagenomic samples

**Figure 3:**
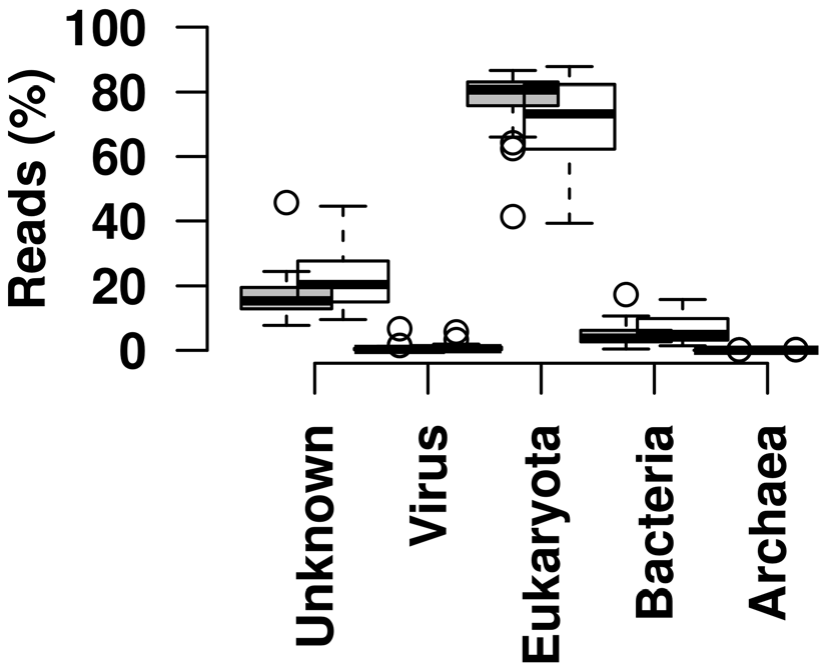
Relative abundance of broad taxonomic categories in metagenomic sequences obtained from nose (grey) and eye swab (white) samples of caribou. Sequences with an unidentified taxonomic category assigned were excluded. Top and bottom of the box indicate the first and third quantile, the band inside the box marks the median. Whiskers extend to the upper and lower quartiles, and outliers are plotted as individual points.

Most taxonomic units of all metagenomic samples of caribou eye and nose swabs were of eukaryotic origin (range 39 to 88%), followed by unknown (8 to 46%) and bacterial origin (0.4 to 17%) (Figure 3). The viral content ranged from 0 (nose swab of animal 28) to 6.6%. There was no significant difference in taxonomic content between eye and nose swabs in these broad categories, however, the taxonomic content of the different samples varied substantially. The nasal swab of animal 26 (adult female) constituted an outlier. This was exemplified by plotting the theoretical quantiles of the normal distribution against the sample quantiles of the compression factors (number of reads divided by the number of taxonomic units after assembly, Table S1 and Figure S2), the duplication levels (defined as the mean percentage of reads that were not unique within a random selection of five times 500 trimmed reads) and the number of filtered taxonomic units containing more than 60% low complexity sequence of all samples (Figure S2). Consequently, 37.9% of the taxonomic units of this sample were filtered as low complexity sequences before homology search, in contrast to a median of 5.5% low complexity sequences for other samples (data not shown). Accordingly, this sample did not contain any identifiable virus sequences.

In all samples with viral content, a large part of the retrieved putative viral sequences were obtained from bacteriophages. Animal 13 (adult male) contained a taxonomic unit composed of two reads that had homology to a RNA-dependent RNA polymerase of a human picobirnavirus (76% identity on nucleotide level). Animal 20 (adult male) contained 7 taxonomic units composed of 11 reads with homology to viruses of the family *Polyomaviridae*. Taxonomic units related to known viruses of the *Parvoviridae* family were detected in eye and nose swabs of animal 2 (adult female), while sequences related to the *Papillomaviridae* family were found in 24 animals (Table S1). Sequences with homology on the amino acid level to viruses of the family *Coronaviridae* were detected in three eye swabs. Further characterization of the sequences related to the four virus families, *Polyoma-*, *Parvo*-, *Papilloma-*, and *Coronaviridae* is described below.

### Papillomaviridae

With a total of 569 reads in 103 taxonomic units of 24 samples, members of the family *Papillomaviridae* were the most prevalent identified mammalian viruses in the caribou samples (Table S1). Papillomaviruses are classified on basis of the phylogenetic distance of the entire L1 sequence to other members of the family, taking into account the genome organization, biology, and pathogenicity. Intergeneric identities range from about 43–62%, interspecies identities from about 55–71%, and intraspecies identities from about 67–88% [28], [29].

**Figure 4:**
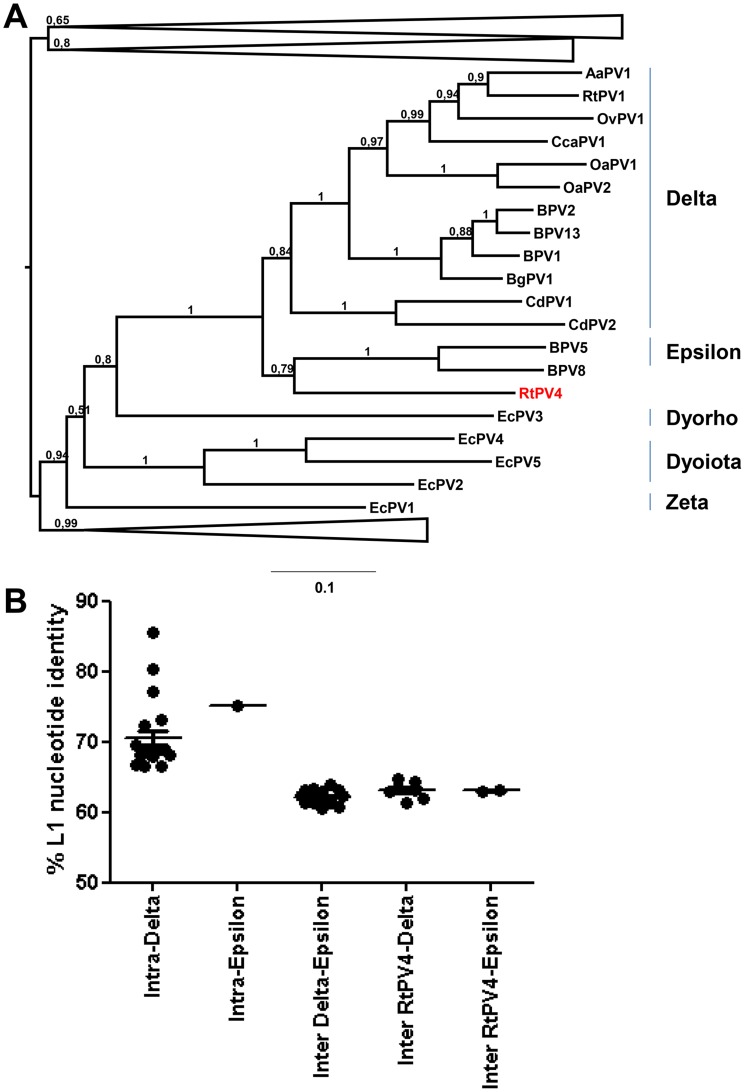
Phylogenetic analysis of RtPV4. PhyML tree with GTR and 100 bootstrap replicates of a nucleotide sequence alignment of L1 genes of caribou papillomavirus RtPV4 from the eye swab of animal 26 and various viruses of the family *Papillomaviridae*. Significant bootstrap values are shown. All sequences were retrieved from PAVE (http://pave.niaid.nih.gov).

Apart from a complete L1 sequence recovered from the eye swab of animal 26, two other large putative papillomavirus contigs were identified in the same sample. Together they covered a total of 7,079 bp of a putative reindeer papillomavirus by 275 reads, called *Rangifer tarandus* papillomavirus RtPV4. The RtPV4 L1 nucleotide sequence of animal 26 was compared to known papillomaviruses for which a full-length L1 sequence was present in PAVE (#http://pave.niaid.nih.gov/#home). A phylogenetic tree was generated for a subset of representative papillomaviruses for visibility (Figure 4A). The most closely related papillomaviruses to RtPV4 were those from the genera *Delta*- and *Epsilonpapillomavirus* with ∼63% nucleotide identity in L1, which is at the border of intergeneric nucleotide identity [28], [29] (Figure 4B). The genome organization was comparable to that of *Delta*- and *Epsionpapillomaviruses* as well. Based on the sequence identity criteria and the sequence identity equidistance of RtPV4 to viruses from the *Delta*- and *Epsilonpapillomavirus* genera, RtPV4 could be designated as a new papillomavirus genus.

In many samples no full L1 sequence was retrieved from eye and/or nose swabs; therefore no official classification was possible. However, taxonomic units from 10 samples that were identified as a fragment of putative L1 proteins were used to estimate the closest relative within the family *Papillomaviridae* (Table 1) by determining their closest homolog. Putative L1 taxonomic units from five nose swabs, but none from eye swabs, had a closest relative with human papillomaviruses with a nucleotide similarity between 75%-100% (not included in Table 1). The remaining five samples (three eye swabs and two nose swabs) from four different animals had in total seven putative L1 sequences (Table 1). Five were most closely related to members of genus *Xipapillomavirus* from cattle (BPV12, BPV10, and BPV11) with 71%–76% nucleotide identity and to *Rangifer tarandus* papillomavirus 2 (RtPv2) with 68%–73% and another with 70% nucleotide identity to *Capra hircus* papillomavirus type 1 (ChPV1) from goat (Table 1). The L1 sequence of animal 26, as described above, was most closely related to members of genus *Delta-* and *Epsilonpapillomavirus*. Of interest, the nasal swab from animal 27 contained at least two different papillomaviruses types (Table 1). In contrast, sequences yielded from nose and eye of animal 19 had both a closest homology with BPV10, suggesting the same infection in nose and eyes.

**Table 1:** Putative partial *Papillomaviridae* L1 sequences identified in four different animals.

Animal number	Swab	L1 gene	Fragment length (bases)	Nucleotide identity %
2	Eye	BPV12 (xi)	411	73
19	Eye	BPV10 (xi)	668/416	72–76
19	Nose	BPV10 (xi)	569	76
26	Eye	BPV2 (delta)	1485	65
27	Nose	ChPV1 (phi)	350	70
27	Nose	BPV11 (xi)	374	71

The different papillomavirus genera are indicated in brackets. L1 genes are typically about 1500 bases in length.

### Parvoviridae

**Figure 5:**
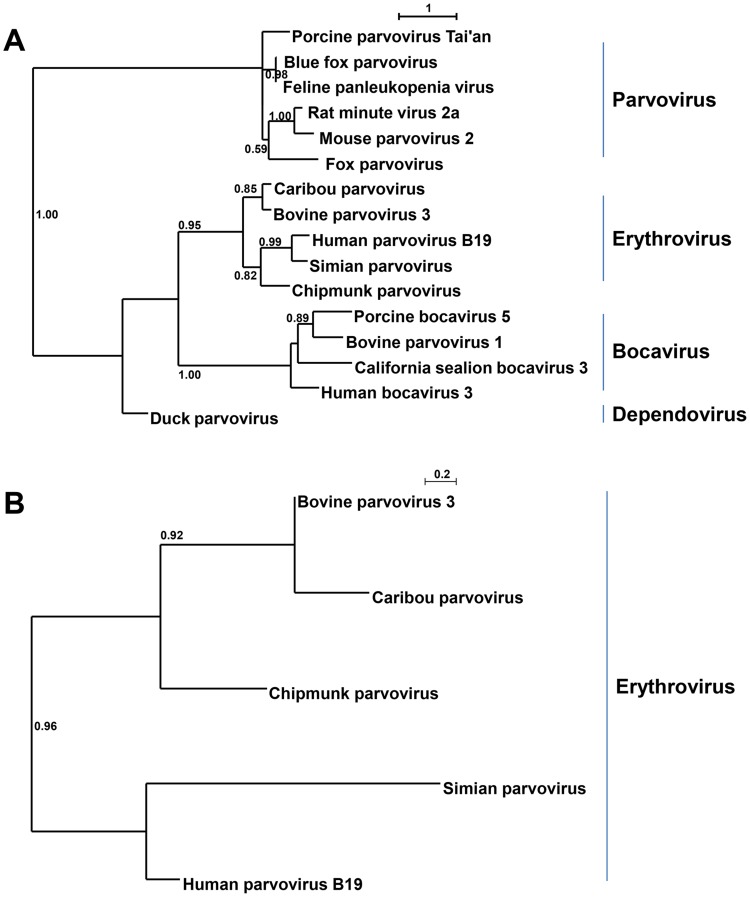
Phylogenetic analysis of caribou parvovirus. (A) PhyML tree with LG and 100 bootstrap replicates of a fragment of the VP2 proteins of caribou parvovirus from the nose swab of animal 2 and various viruses of the subfamily *Parvovirinae*, corresponding to VP2 amino acids 737 to 914 in Bovine parvovirus 3 VP2 (AF406967). (B) PhyML tree with LG and 100 bootstrap replicates of the amino acid sequence of a fragment of the NS1 genes of caribou parvovirus from animal 2 and various viruses of the genus *Erythrovirus*, corresponding to amino acids 235 to 305 in Bovine parvovirus 3 NS1 (AF406967). Significant bootstrap values are shown. See Table S2 for accession numbers.

*Erythrovirus* is a genus in the family *Parvoviridae* which consists of small, icosahedral, non-enveloped viruses. So far, four species (Human parvovirus B19, pig-tailed macaque parvovirus, Rhesus macaque parvovirus and Simian parvovirus) and two tentative members have been identified. In eye and nose swabs of animal 2 (adult female), evidence was found for the presence of a parvovirus of the family *Parvoviridae*. A total of 11 taxonomic units, composed of 23 reads, showed either on nucleotide or amino acid level homology to bovine parvovirus 3 (accession number AAL09674), a species in the genus *Erythrovirus* [30]. The contigs were mainly covering the putative capsid protein genes with a nucleotide identity of 70%–75% to the corresponding genome regions of bovine parvovirus 3. A phylogenetic tree of a fragment of the capsid protein (amino acids 737 to 914 in Bovine parvovirus 3 VP2 (AF406967)) showed that the caribou parvovirus clustered closely with bovine parvovirus type 3 and other members of the genus *Erythrovirus* (Figure 5A). A similar clustering was observed upon phylogenetic analysis of a fragment of the NS1 protein (Figure 5B).

### Coronaviridae

**Figure 6:**
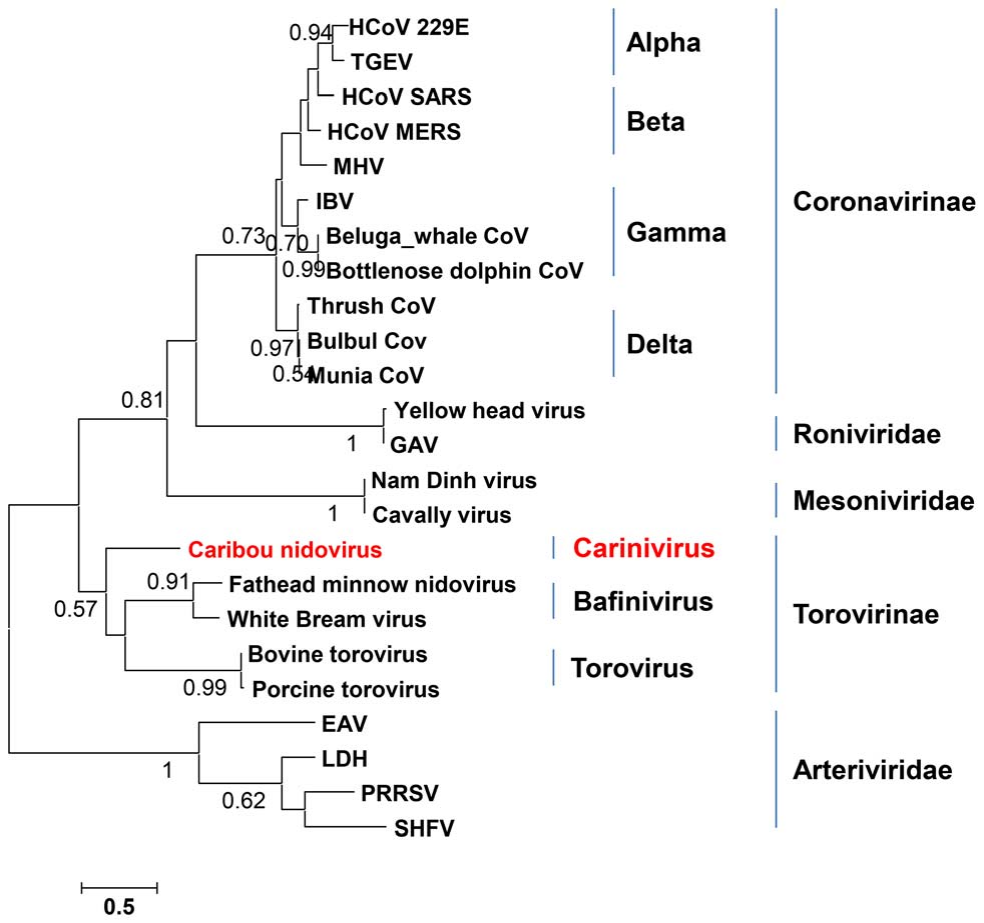
Phylogenetic analysis of caribou nidovirus. Phylogenetic Maximum Likelihood tree with WAG with Freqs. and Gamma distributed with Invariant sites (G+I) parameters of an amino acid sequence alignment of a helicase fragment of an eye swab from animal 9 and the corresponding nidovirus sequences (corresponding to positions 5667 to 5916 in White Bream virus). Bootstrap values (100 replicates) are shown. See Table S2 for accession numbers.

The order *Nidovirales* is composed of the families *Corona-*, *Arteri-*, *Roni-*, and *Mesoniviridae* and contains large (up to 32 kb) positive-stranded RNA viruses. Within the family *Coronaviridae*, two subfamilies, *Coronavirinae* and *Torovirinae* have been proposed in which four and two genera have been classified, respectively. A total of 11 taxonomic units composed of 37 reads in three eye swabs of animals 9, 27, and 30, all adult females, showed homology to members of the family *Coronaviridae*, subfamily *Torovirinae* (Table S1). A meta-assembly from all three eye swabs uncovered parts of the putative polyprotein 1ab (pp1ab) with homology to either *Bafiniviruses* (amino acid identity range from 37%–50%) or to *Toroviruses* (range 36%–60%). A phylogenetic tree was constructed with a partial helicase amino acid sequence from animal 9 and the corresponding sequences of other *Nidoviruses* (Figure 6). The phylogenetic tree showed that the sequence identified in caribou 9 clustered between *Bafinivirus* and *Torovirus*, suggesting that it is a divergent species potentially representing a new genus, which we tentatively called *Carinivirus*. Efforts to obtain more viral sequences were not successful, due to low viral titers in the samples and a finite volume of samples.

### Polyomaviridae

**Figure 7:**
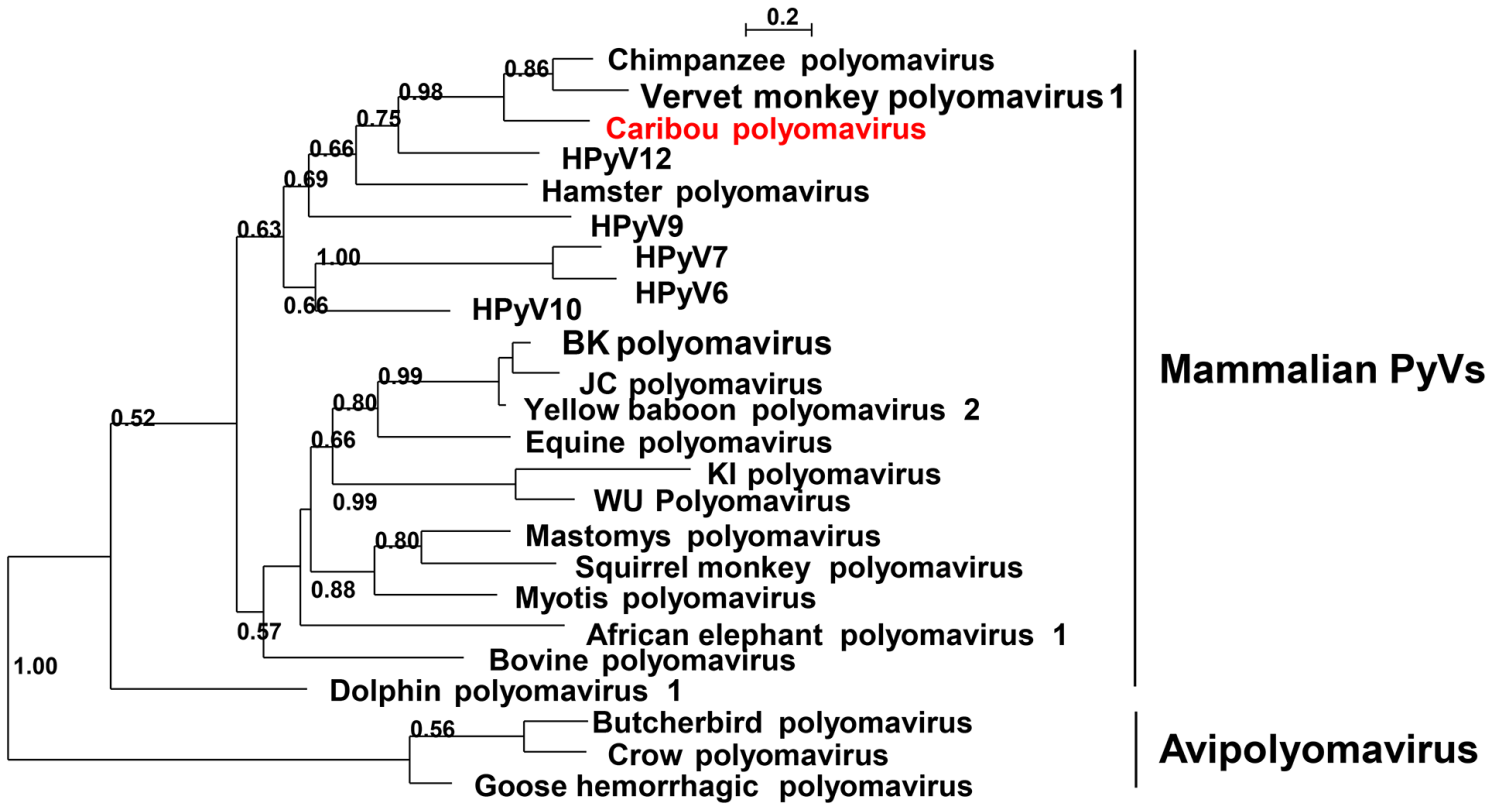
Phylogenetic analysis of caribou polyomavirus. PhyML tree with LG and 100 bootstrap replicates of a fragment of the large T antigen of caribou polyomavirus from the nose swab of animal 20 and various viruses of the family *Polyomaviridae* (corresponding to large T antigen amino acids 305 to 390 of Chimpanzee polyomavirus (CBX23452)). Significant bootstrap values are shown. See Table S2 for accession numbers. Mammalian and avian polyomaviruses (PyVs) are indicated.

Polyomaviruses (family *Polyomaviridae*) are small circular double-stranded DNA viruses of approximately 5000 bp. Most mammalian polyomaviruses, which are highly host specific, cause subclinical infections with life-long persistence in their natural immunocompetent hosts. However, in immunocompromised individuals, they can cause disease [31]. The International Committee on Taxonomy of Viruses officially lists thirteen species of polyomaviruses. Three genera, two containing mammalian viruses (genus *Orthopolyomavirus*, and genus *Wukipolyomavirus*) and one containing avian viruses (genus *Avipolyomavirus*) have been proposed [31]. Animal 20 (adult male) contained viral sequences with homology to viruses of the family *Polyomaviridae* in both eye and nose swabs. A phylogenetic tree of a fragment of the large T antigen protein showed that the caribou polyomavirus clustered closely with chimpanzee and vervet monkey polyomaviruses and displayed ∼62.5% amino acid identity to these viruses (Figure 7).

## Discussion

Pathogen surveillance in domestic animals and wildlife is a crucial aspect in trying to understand the etiology of disease, their impact on individual health as well as on populations over time, but also for preparedness for emerging viral diseases, as most (re-)emerging viral infections originate from an animal reservoir, as exemplified by the outbreaks of SARS coronavirus and H7N7 influenza A virus in which pathogen surveillance in animals, or its integration with public health, has faltered [32]. The advent of high-throughput sequencing and advanced bioinformatic analyses have enabled more substantiated identification and phylogeny studies of viruses present in wildlife reservoirs in recent years.

In this study, the presence of viruses in free-ranging caribou was studied by a metagenomic survey of swab samples obtained from the mucosa of the eye and nose. The obtained metagenomes were subjected to different assembly algorithms prior to homology search to optimize the chance of identifying viral species in the metagenome dataset. The assembly strategy and parameters should ideally be selected based on the focus and conditions of a study, as a study for comparison of different assemblers for viral metagenomes recently showed [33] and the choice might be different even for similar projects. The comparison of different assembly strategies applied here involved a limited number of assemblers with single parameter sets. In our hands, the exhaustive iterative assembly approach seemed optimal and was added to an automated virus discovery pipeline. Iterative assembly is also used by PRICE [34], an assembler for components of metagenomes in which an input target sequence is required. Our approach is completely *de novo* and unsupervised. It ensures optimal assembly in our comparative analysis with Newbler, CAP3, Mira, and CLC Genomics Workbench, either in terms of reliability and/or compression of the output data. This approach therefore facilitates subsequent homology searches, as longer, more reliable queries are more likely to yield functional or taxonomic assignments by exhibiting homology to a previously characterized and deposited sequence. The strategy of iterative assembly applied in this study is easily transferable to different kinds of algorithms, assemblers, and parameters, or a mix thereof, depending on the nature of the data. The mapping step after convergence of the assembly ensures not only the validity of the assemblies, but also allows tracing the origin of single sequencing reads which allows an estimation of abundance of viral and other sequences. In the caribou cohort, consisting of presumably healthy animals sampled on their way south during their seasonal migration, up to 6.6% of the sequence reads were of viral origin in each sample, of which most were derived from bacteriophages.

Members of the family *Papillomaviridae* were the most prevalent identified mammalian viruses in the caribou samples. L1 is the most conserved region among papillomaviruses and according to the current genus classification and its equidistance from *Delta*- and *Epsilonpapillomaviruses*, RtPV4 from an eye swab of animal 26 could be designated as a new papillomavirus genus of the family *Papillomaviridae*. Interestingly, RtPV4 from *Rangifer tarandus granti* in Alaska is more closely related to bovine papillomaviruses 1 and 2 in the L1 gene sequence, as compared to RtPV1 from Swedish reindeer (*Rangifer t. tarandus*) and a papillomavirus from American white-tailed deer (*Odocoileus virginianus*). In humans, papillomaviruses display high genetic diversity, with over 140 recognized types. For animals, however, more than ten papillomavirus types per host species have so far only been discovered in well studied vertebrates, such as cattle, dogs and macaques [35]. In reindeer, two types are described and so far genomically characterized in the genera *Delta*- and *Xipapillomavirus* [5], [36]. This study revealed the presence of at least three different papillomavirus types in caribou. This suggests that members of the family *Papillomaviridae* in reindeer are more diverse than currently acknowledged. Also human papillomaviruses were identified in this study, mainly in nose swabs, and they are likely to originate from contact with human skin. In this case, they assumingly originate from the sampling team. Although papillomaviruses have been identified a few times in reindeer [4], [37], not much knowledge exists on the etiology of papillomatosis in reindeer. The disease is observed from time to time, both in wild reindeer and semi-domesticated reindeer, although it is not considered as a common disease. Generalized papillomatosis may contribute to severe conditions and mortality of affected individuals, and the clinical impact on the population or herd level is expected to be restricted, but the impact of papillomavirus infections on reindeer in general should be further addressed.

Evidence for the presence of a member of the family *Parvoviridae* was found in caribou 2. Dependent on the species, clinical signs following infection with a parvovirus can be absent or severe, including death [38]. Caribou parvovirus capsid sequences showed 70–75% nucleotide identity to bovine parvovirus 3. Bovine parvovirus 3 would be a member of the species ungulate erythroparvovirus 1 as recently proposed in a revision of the *Parvoviridae* taxonomy [30]. The proposal will decrease species identity criteria significantly, requiring viruses within a species to encode complete NS1 proteins that show 85% amino acid sequence identity while diverging by 15% from viruses in other species [30]. Our data suggest that the caribou parvovirus would constitute a divergent species in the genus *Erythrovirus*, as it displays ∼70% amino acid identity in a small fragment of NS1 to its closest relative bovine parvovirus 3.

The criteria utilized in creating new taxa for *Polyomaviridae* will include biological properties as well as genetic relationships. These criteria will include (i) host range, (ii) genetic repertoire and (iii) DNA sequence identity over the whole genome (81–84% for species) [31]. The caribou polyomavirus was most closely related to chimpanzee polyomavirus in a parial large T antigen fragment and would likely constitute a new polyomavirus species. Polyomaviruses often persist as latent infections in a host without causing disease, but are potentially oncogenic [39]. The caribou polyomavirus was detected in samples from both eye and nose mucosa, which is similar to the recently described equine polyomavirus that was detected in eye samples from horses with clinical uveitis [40]. Whether the caribou polyomavirus is involved in causing disease in caribou remains to be determined.

Possibly the most interesting observation is the presence of viral sequences with homology to bafini- and toroviruses in eye swabs of 3 apparently healthy caribou. The observed homology of sequence fragments of this caribou nidovirus with other viruses in the order *Nidovirales* is very low and phylogenetic analysis of a partial helicase protein fragment suggest that the caribou nidovirus is a divergent species potentially representing a new genus in the family *Coronaviridae*, subfamily *Torovirinae*, which we tentatively called *Carinivirus* for Caribou-associated nidovirus. Efforts to obtain more viral sequences were not successful, due to low viral titers in the samples and a restricted sample volume. Coronaviruses are thought to predominantly infect epithelia and are mostly associated with respiratory and gastrointestinal infections [41]. It is of note that the caribou nidovirus sequences were detected in eye swabs of caribou only. The ability of coronaviruses to replicate in the eye has been described previously, where mouse hepatitis virus A59 was shown to induce optic neuritis in an experimental animal model [42], [43] and feline coronaviruses were capable of causing uveitis [44]. In addition, the presence of SARS coronavirus in tears has been reported [45].

The discovery of new papilloma-, polyoma-, corona-, and parvoviruses from caribou as described here is an example of the required expansion of our knowledge of the virus diversity present in wildlife, as well as the potential transmission to and infection of other wildlife species, pets and production animals. The WACH population has declined since its recorded peak in 2003. Many different conditions, such as predators, weather and climatic conditions and diseases, may partly explain such fluctuations. Caribou individuals reaching the Kobuk River on their way south to their winter range may be considered as “winners”, being healthy and able to conduct such a strenuous seasonal migration. It is thus possible that individuals of the same population, that do not make it to the winter ranges, may suffer from similar infections and that they are also affected clinically. To search for associations between the virus infections detected during this work and potential disease and impact on individuals and the population, samples should be obtained from animals found dead or from individuals that, for some reason, do not migrate with the main herd to the winter pastures. The caribou monitored in this study appeared healthy and the disease potential of the newly identified viruses therefore remains to be determined.

## Supporting Information

Figure S1 **Reduction of metagenomic datasets as achieved by exhaustive iterative assembly for eight randomly selected samples: Trimmed reads (“Trimmed”) were subjected to initial assembly with GSAssembler (“Newbler”), followed by iterative rounds of assembly with CAP3 until convergence (“CAP3-first” to “CAP3-last”) as described in the text.** Datasets were further reduced by mapping of trimmed reads to taxonomic units and size selection of units longer than 75 bases (“Mapped”) and filtering of sequences composed of more than 60% low-complexity sequences (“Filtered”). Indicated are the number of taxonomic units, i.e. the sum of contigs and singletons.
(TIF)

Figure S2 **Theoretical quantiles of a normal distribution plotted against sample quantiles for (A) sequence compression factor by assembly, (B) duplication levels, and (C) low complexity sequences in taxonomic units in 78 metagenomic samples.** The most prominent outlier was excluded from further analysis.
(TIF)

Table S1 Sample collection of caribou (*Rangifer tarandus granti*) from the Western Arctic herd (Animals 1 to 39), Alaska, and reads and taxonomic units (Tax. Un.) obtained by next-generation sequencing and exhaustive iterative assembly. * excluded from analysis.
(DOCX)

Table S2 Virus sequences and their accession number used for phylogenetic trees in this study.
(DOCX)

## References

[1] das Neves C, Mork T, Thiry J, Godfroid J, Rimstad E, et al (2009) Cervid herpesvirus 2 experimentally reactivated in reindeer can produce generalized viremia and abortion. Virus Res 145: 321–328. 19699769 10.1016/j.virusres.2009.08.002

[2] Dau J (2007) Units 21D, 22A, 22B, 22C, 22D, 22E, 23, 24 and 26A caribou management report. Book Units 21D, 22A, 22B, 22C, 22D, 22E, 23, 24 and 26A caribou management report Fairbanks, Alaska, Alaska Department of Fish and Game: 174 – 231.

[3] Evans AL, das Neves CG, Finstad GF, Beckmen KB, Skjerve E, et al (2012) Evidence of alphaherpesvirus infections in Alaskan caribou and reindeer. BMC Vet Res 8: 5. 22243919 10.1186/1746-6148-8-5 PMC3274481

[4] Evans A, Bey R, Schoster J, Gaarder J, Finstad G (2008) Preliminary studies on the etiology of keratoconjunctivitis in reindeer (Rangifer tarandus tarandus) calves in Alaska. J Wildl Dis 44: 1051–1055. 18957667 10.7589/0090-3558-44.4.1051

[5] Smits SL, Schapendonk CM, van Leeuwen M, Kuiken T, Bodewes R, et al (2013) Identification and Characterization of Two Novel Viruses in Ocular Infections in Reindeer. PloS one 8: e69711. 23874987 10.1371/journal.pone.0069711 PMC3713034

[6] Bodewes R, van der Giessen J, Haagmans BL, Osterhaus AD, Smits SL (2013) Identification of multiple novel viruses, including a parvovirus and a hepevirus, in feces of red foxes. J Virol 87: 7758–7764. 23616657 10.1128/JVI.00568-13 PMC3700315

[7] Mokili JL, Rohwer F, Dutilh BE (2012) Metagenomics and future perspectives in virus discovery. Curr Opin Virol 2: 63–77. 22440968 10.1016/j.coviro.2011.12.004 PMC7102772

[8] Venter JC, Remington K, Heidelberg JF, Halpern AL, Rusch D, et al (2004) Environmental genome shotgun sequencing of the Sargasso Sea. Science 304: 66–74. 15001713 10.1126/science.1093857

[9] Kunin V, Copeland A, Lapidus A, Mavromatis K, Hugenholtz P (2008) A bioinformatician's guide to metagenomics. Microbiology and Molecular Biology Reviews 72: 557–578. 19052320 10.1128/MMBR.00009-08 PMC2593568

[10] Hall RJ, Wang J, Todd AK, Bissielo AB, Yen S, et al (2014) Evaluation of rapid and simple techniques for the enrichment of viruses prior to metagenomic virus discovery. Journal of virological methods 195: 194–204. 24036074 10.1016/j.jviromet.2013.08.035 PMC7113663

[11] Rosseel T, Van Borm S, Vandenbussche F, Hoffmann B, van den Berg T, et al (2013) The Origin of Biased Sequence Depth in Sequence-Independent Nucleic Acid Amplification and Optimization for Efficient Massive Parallel Sequencing. PloS one 8: e76144. 24086702 10.1371/journal.pone.0076144 PMC3784409

[12] Hansen KD, Brenner SE, Dudoit S (2010) Biases in Illumina transcriptome sequencing caused by random hexamer priming. Nucleic acids research 38: e131. 20395217 10.1093/nar/gkq224 PMC2896536

[13] Karlsson OE, Hansen T, Knutsson R, Löfström C, Granberg F, et al (2013) Metagenomic detection methods in biopreparedness outbreak scenarios. Biosecurity and bioterrorism: biodefense strategy, practice, and science 11: S146–S157. 10.1089/bsp.2012.0077 23971800

[14] Ross MG, Russ C, Costello M, Hollinger A, Lennon NJ, et al (2013) Characterizing and measuring bias in sequence data. Genome Biol 14: R51. 23718773 10.1186/gb-2013-14-5-r51 PMC4053816

[15] Wendl MC, Kota K, Weinstock GM, Mitreva M (2012) Coverage theories for metagenomic DNA sequencing based on a generalization of Stevens' theorem. Journal of Mathematical Biology: 1–21. 10.1007/s00285-012-0586-x PMC3795925 22965653

[16] Breitbart M, Hewson I, Felts B, Mahaffy JM, Nulton J, et al (2003) Metagenomic analyses of an uncultured viral community from human feces. Journal of bacteriology 185: 6220–6223. 14526037 10.1128/JB.185.20.6220-6223.2003 PMC225035

[17] Mavromatis K, Ivanova N, Barry K, Shapiro H, Goltsman E, et al (2007) Use of simulated data sets to evaluate the fidelity of metagenomic processing methods. Nature methods 4: 495–500. 17468765 10.1038/nmeth1043

[18] Huang X, Madan A (1999) CAP3: A DNA sequence assembly program. Genome research 9: 868–877. 10508846 10.1101/gr.9.9.868 PMC310812

[19] Pop M (2009) Genome assembly reborn: recent computational challenges. Briefings in bioinformatics 10: 354–366. 19482960 10.1093/bib/bbp026 PMC2691937

[20] Margulies M, Egholm M, Altman WE, Attiya S, Bader JS, et al (2005) Genome sequencing in microfabricated high-density picolitre reactors. Nature 437: 376–380. 16056220 10.1038/nature03959 PMC1464427

[21] Denisov G, Walenz B, Halpern AL, Miller J, Axelrod N, et al (2008) Consensus generation and variant detection by Celera Assembler. Bioinformatics 24: 1035–1040. 18321888 10.1093/bioinformatics/btn074

[22] Batzoglou S, Jaffe DB, Stanley K, Butler J, Gnerre S, et al (2002) ARACHNE: a whole-genome shotgun assembler. Genome research 12: 177–189. 11779843 10.1101/gr.208902 PMC155255

[23] van den Brand JM, van Leeuwen M, Schapendonk CM, Simon JH, Haagmans BL, et al (2012) Metagenomic analysis of the viral flora of pine marten and European badger feces. Journal of virology 86: 2360–2365. 22171250 10.1128/JVI.06373-11 PMC3302375

[24] van Leeuwen M, Williams MM, Koraka P, Simon JH, Smits SL, et al (2010) Human picobirnaviruses identified by molecular screening of diarrhea samples. Journal of clinical microbiology 48: 1787–1794. 20335418 10.1128/JCM.02452-09 PMC2863890

[25] Richter DC, Ott F, Auch AF, Schmid R, Huson DH (2008) MetaSim—A sequencing simulator for genomics and metagenomics. PloS one 3: e3373. 18841204 10.1371/journal.pone.0003373 PMC2556396

[26] Chevreux B, Wetter T, Suhai S (1999) Genome sequence assembly using trace signals and additional sequence information. Computer Science and Biology: Proceedings of the German Conference on Bioinformatics 99: 45–56.

[27] Haagmans BL, Al Dhahiry SH, Reusken CB, Raj VS, Galiano M, et al (2014) Middle East respiratory syndrome coronavirus in dromedary camels: an outbreak investigation. Lancet Infect Dis 14: 140–145. 24355866 10.1016/S1473-3099(13)70690-X PMC7106553

[28] Bernard H-U, Burk RD, Chen Z, van Doorslaer K, Hausen Hz, et al (2010) Classification of papillomaviruses (PVs) based on 189 PV types and proposal of taxonomic amendments. Virology 401: 70–79. 20206957 10.1016/j.virol.2010.02.002 PMC3400342

[29] De Villiers E-M, Fauquet C, Broker TR, Bernard H-U, zur Hausen H (2004) Classification of papillomaviruses. Virology 324: 17–27. 15183049 10.1016/j.virol.2004.03.033

[30] Cotmore SF, Agbandje-McKenna M, Chiorini JA, Mukha DV, Pintel DJ, et al.. (2013) The family Parvoviridae. Archives of virology: 1–9. 10.1007/s00705-013-1914-1 PMC4013247 24212889

[31] Johne R, Buck CB, Allander T, Atwood WJ, Garcea RL, et al (2011) Taxonomical developments in the family Polyomaviridae. Arch Virol 156: 1627–1634. 21562881 10.1007/s00705-011-1008-x PMC3815707

[32] Kuiken T, Leighton FA, Fouchier RA, LeDuc JW, Peiris JS, et al (2005) Public health. Pathogen surveillance in animals. Science 309: 1680–1681. 16150997 10.1126/science.1113310

[33] Vázquez-Castellanos JF, García-López R, Pérez-Brocal V, Pignatelli M, Moya A (2014) Comparison of different assembly and annotation tools on analysis of simulated viral metagenomic communities in the gut. BMC Genomics 15: 37. 24438450 10.1186/1471-2164-15-37 PMC3901335

[34] Ruby JG, Bellare P, DeRisi JL (2013) PRICE: Software for the Targeted Assembly of Components of (Meta) Genomic Sequence Data. G3 (Betheseda) 3: 865–880. 10.1534/g3.113.005967 PMC3656733 23550143

[35] Rector A, Van Ranst M (2013) Animal papillomaviruses. Virology 445: 213–223. 23711385 10.1016/j.virol.2013.05.007

[36] Terai M, DeSalle R, Burk RD (2002) Lack of canonical E6 and E7 open reading frames in bird papillomaviruses: Fringilla coelebs papillomavirus and Psittacus erithacus timneh papillomavirus. Journal of virology 76: 10020–10023. 12208979 10.1128/JVI.76.19.10020-10023.2002 PMC136527

[37] Eriksson A, Stewart AC, Moreno-Lopez J, Pettersson U (1994) The genomes of the animal papillomaviruses European elk papillomavirus, deer papillomavirus, and reindeer papillomavirus contain a novel transforming gene (E9) near the early polyadenylation site. J Virol 68: 8365–8373. 7966628 10.1128/jvi.68.12.8365-8373.1994 PMC237305

[38] Berns KI, Parrish CR (2007) *Parvoviridae*. In: Knipe DM, Howley PM, editors. Fields virology, 5th ed. Philadelphia, PA: Lippincott Williams & Wilkins Publishers. pp. 2437–2477.

[39] Delbue S, Comar M, Ferrante P (2012) Review on the relationship between human polyomaviruses-associated tumors and host immune system. Clin Dev Immunol 2012: 542092. 22489251 10.1155/2012/542092 PMC3318214

[40] Renshaw RW, Wise AG, Maes RK, Dubovi EJ (2012) Complete genome sequence of a polyomavirus isolated from horses. J Virol 86: 8903. 22843861 10.1128/JVI.01261-12 PMC3421721

[41] de Groot RJ, Baker SC, Baric R, Enjuanes L, Gorbalenya AE, et al. (2011) Family *Coronaviridae*. In: King AM, Adams MJ, Lefkowitz EJ, Carstens EB, editors. Virus taxonomy: IXth report of the International Committee on Taxonomy of Viruses: pp. 806–828.

[42] Shindler KS, Kenyon LC, Dutt M, Hingley ST, Sarma JD (2008) Experimental optic neuritis induced by a demyelinating strain of mouse hepatitis virus. Journal of virology 82: 8882–8886. 18579591 10.1128/JVI.00920-08 PMC2519666

[43] Robbins S, Hamel C, Detrick B, Hooks J (1990) Murine coronavirus induces an acute and long-lasting disease of the retina. Laboratory investigation; a journal of technical methods and pathology 62: 417–426. 2159082

[44] Stiles J (2011) Bartonellosis in cats: a role in uveitis? Veterinary ophthalmology 14: 9–14. 21923819 10.1111/j.1463-5224.2011.00901.x

[45] Loon S, Teoh S, Oon L, Se-Thoe S, Ling A, et al (2004) The severe acute respiratory syndrome coronavirus in tears. British journal of ophthalmology 88: 861–863. 15205225 10.1136/bjo.2003.035931 PMC1772213

