# Supplementary material for: Metagenomic Survey for Viruses in Western Arctic Caribou, Alaska, through Iterative Assembly of Taxonomic Units
Source: PLoS One. 2014 Aug 20;9(8):e105227. doi: 10.1371/journal.pone.0105227 (PMC4139337; doi:10.1371/journal.pone.0105227)
Supplement: Table S1 — Sample collection of caribou (Rangifer tarandus granti) from the Western Arctic herd (Animals 1 to 39), Alaska, and reads and taxonomic units (Tax. Un.) obtained by next-generation sequencing and exhaustive iterative assembly. * excluded from analysis. (DOCX) [file pone.0105227.s003.docx]

**Table S1.** Sample collection of caribou (*Rangifer tarandus granti*) from the Western Arctic herd (Animals 1 to 39), Alaska, and reads and taxonomic units (Tax. Un.) obtained by next-generation sequencing and exhaustive iterative assembly. * excluded from analysis.

| **Animal** | **Sample** | **Reads** | **Tax. Un.** | **Mammalian virus** | **Sample** | **Reads** | **Tax. Un.** | **Mammalian virus** |
| --- | --- | --- | --- | --- | --- | --- | --- | --- |
| 1 | Eye | 11379 | 5354 |  | Nose | 9832 | 4685 |  |
| 2 | Eye | 8587 | 4298 | *Parvoviridae* | Nose | 13503 | 6568 | *Parvoviridae* |
| 3 | Eye | 664 | 301 |  | Nose | 10841 | 4749 |  |
| 4 | Eye | 9557 | 5287 |  | Nose | 9113 | 4136 |  |
| 5 | Eye | 9625 | 3613 |  | Nose | 8358 | 3543 |  |
| 6 | Eye | 32066 | 14725 |  | Nose | 9863 | 4627 |  |
| 7 | Eye | 12029 | 5785 |  | Nose | 12496 | 5925 |  |
| 8 | Eye | 20182 | 9583 | *Papillomaviridae* | Nose | 12348 | 4410 | *Papillomaviridae* |
| 9 | Eye | 15298 | 8002 | *Coronaviridae* | Nose | 16555 | 6906 |  |
| 10 | Eye | 11977 | 5677 |  | Nose | 18034 | 7271 | *Papillomaviridae* |
| 11 | Eye | 10698 | 5436 |  | Nose | 6399 | 2690 |  |
| 12 | Eye | 18274 | 7786 | *Papillomaviridae* | Nose | 9085 | 4247 | *Papillomaviridae* |
| 13 | Eye | 16110 | 6960 | *Picobirnaviridae* | Nose | 11336 | 4586 | *Picobirnaviridae* |
|  |  |  |  | *Papillomaviridae* |  |  |  |  |
| 14 | Eye | 10515 | 5274 |  | Nose | 14588 | 5744 | *Papillomaviridae* |
| 15 | Eye | 1458 | 643 |  | Nose | 14224 | 5464 |  |
| 16 | Eye | 11154 | 5863 |  | Nose | 14873 | 5523 |  |
| 17 | Eye | 16590 | 8842 | *Papillomaviridae* | Nose | 17294 | 6969 | *Papillomaviridae* |
| 18 | Eye | 27368 | 12320 |  | Nose | 11950 | 4868 |  |
| 19 | Eye | 19293 | 8452 | *Papillomaviridae* | Nose | 6609 | 2988 | *Papillomaviridae* |
| 20 | Eye | 20327 | 8987 | *Polyomaviridae* | Nose | 17257 | 6934 | *Polyomaviridae* |
|  |  |  |  | *Papillomaviridae* |  |  |  | *Papillomaviridae* |
| 21 | Eye | 13974 | 7031 |  | Nose | 15955 | 6511 |  |
| 22 | Eye | 20725 | 10666 |  | Nose | 13043 | 5390 | *Papillomaviridae* |
| 23 | Eye | 8416 | 3972 |  | Nose | 6144 | 2261 |  |
| 24 | Eye | 12141 | 5715 |  | Nose | 14391 | 6090 |  |
| 25 | Eye | 13822 | 5970 |  | Nose | 8715 | 3837 |  |
| 26 | Eye | 13844 | 6496 | *Papillomaviridae* | Nose* | 15836* | 211* |  |
| 27 | Eye | 4934 | 2039 | *Papillomaviridae* | Nose | 16981 | 5179 | *Papillomaviridae* |
|  |  |  |  | *Coronaviridae* |  |  |  |  |
| 28 | Eye | 13747 | 6144 |  | Nose | 15160 | 4388 | *Papillomaviridae* |
| 29 | Eye | 15381 | 6656 |  | Nose | 21536 | 11782 | *Papillomaviridae* |
| 30 | Eye | 20957 | 8286 | *Coronaviridae* | Nose | 43702 | 19533 | *Papillomaviridae* |
| 31 | Eye | 15342 | 6216 | *Papillomaviridae* | Nose | 25971 | 10107 |  |
| 32 | Eye | 14562 | 6503 |  | Nose | 31120 | 12241 |  |
| 33 | Eye | 15742 | 7842 | *Papillomaviridae* | Nose | 8425 | 4244 | *Papillomaviridae* |
| 34 | Eye | 13228 | 5831 |  | Nose | 18120 | 7964 |  |
| 35 | Eye | 17022 | 8571 |  | Nose | 24674 | 10737 |  |
| 36 | Eye | 16515 | 7865 | *Papillomaviridae* | Nose | 38991 | 14612 |  |
| 37 | Eye | 18381 | 8016 |  | Nose | 35651 | 13834 |  |
| 38 | Eye | 13145 | 6433 |  | Nose | 26919 | 12543 |  |
| 39 | Eye | 6044 | 2720 |  | 11924 | 28582 | 11924 |  |
