# Supplementary material for: Metagenomic Survey for Viruses in Western Arctic Caribou, Alaska, through Iterative Assembly of Taxonomic Units
Source: PLoS One. 2014 Aug 20;9(8):e105227. doi: 10.1371/journal.pone.0105227 (PMC4139337; doi:10.1371/journal.pone.0105227)
Supplement: Table S2 — Virus sequences and their accession number used for phylogenetic trees in this study. (DOCX) [file pone.0105227.s004.docx]

**Table S2.** Virus sequences and their accession number used for phylogenetic trees in this study.

| **Virus family** | **Virus name** | **Accession number** |
| --- | --- | --- |
| Parvoviridae | Feline parvovirus V142 | AB054225 |
| Parvoviridae | Blue fox parvovirus | EU698028 |
| Parvoviridae | Porcine parvovirus Tai’an | FJ853421 |
| Parvoviridae | Bovine parvovirus 1 | DQ335247 |
| Parvoviridae | Rat minute virus 2a | EF029111 |
| Parvoviridae | Mouse parvovirus 2 | NC_008186 |
| Parvoviridae | Fox parvovirus | KC692368 |
| Parvoviridae | Simian parvovirus | U26342 |
| Parvoviridae | B19 parvovirus | NC_000883 |
| Parvoviridae | Duck parvovirus | NC_006147 |
| Parvoviridae | Porcine bocavirus 5 | JN831651 |
| Parvoviridae | Human bocavirus 3 | HM132056 |
| Parvoviridae | Bovine parvovirus 3 | AF406967 |
| Parvoviridae | Chipmunk parvovirus | GQ200736 |
| Parvoviridae | Calif. sea lion bocavirus | JN420365 |
| Nidovirales | White bream virus | NC_008516 |
| Nidovirales | Porcine torovirus | NC_022787 |
| Nidovirales | Bovine torovirus | NC_007447 |
| Nidovirales | Fathead minnow nidovirus | GU002364 |
| Nidovirales | Simian hemorrhagic fever virus (SHFV) | NC_003092 |
| Nidovirales | Infectious bronchitis virus (IBV) | KC008600 |
| Nidovirales | Murine hepatitis virus (MHV) | AY700211 |
| Nidovirales | SARS coronavirus (HCoV SARS) | NC_004718 |
| Nidovirales | Yellow head virus | ACA21302 |
| Nidovirales | Cavally virus | YP_004598981 |
| Nidovirales | Porcine reproductive and respiratory virus (PRRSV) | AGN92871 |
| Nidovirales | Nam Dinh virus | ABG02430 |
| Nidovirales | Gill-associated virus (GAV) | YP_001661452 |
| Nidovirales | Human coronavirus 229E (HCoV 229E) | AFR79255 |
| Nidovirales | Munia coronavirus | YP_002308505 |
| Nidovirales | Thrush coronavirus | YP_002308496 |
| Nidovirales | Bulbul coronavirus | ACJ2043 |
| Nidovirales | Transmissible gastroenteritis virus (TGEV) | ABG89333 |
| Nidovirales | Lactate dehydrogenase-elevating virus (LDH) | AAA74103 |
| Nidovirales | Equine arteritis virus (EAV) | P19811 |
| Nidovirales | Beluga whale coronavirus | YP_001876435 |
| Nidovirales | Bottlenose dolphin coronavirus | AHB63507 |
| Nidovirales | Human coronavirus MERS (HCoV MERS) | AFS88944 |
| Polyomaviridae | Bovine polyomavirus | NP_040788 |
| Polyomaviridae | WU polyomavirus | YP_001285488 |
| Polyomaviridae | KI polyomavirus | YP_001111259 |
| Polyomaviridae | Yellow baboon polyomavirus | BAM71853 |
| Polyomaviridae | BK polyomavirus | AEK21506 |
| Polyomaviridae | JC polyomavirus | BAB93105 |
| Polyomaviridae | Equine polyomavirus | YP_006383692 |
| Polyomaviridae | Myotis polyomavirus | YP_002261489 |
| Polyomaviridae | Mastomys polyomavirus | BAJ53087 |
| Polyomaviridae | Squirrel monkey polyomavirus | YP_001531349 |
| Polyomaviridae | African elephant polyomavirus 1 | YP_008603286 |
| Polyomaviridae | HPyV10 | AFN43007 |
| Polyomaviridae | HPyV6 | YP_003848919 |
| Polyomaviridae | HPyV7 | YP_003848924 |
| Polyomaviridae | Hamster polyomavirus | NP_056730 |
| Polyomaviridae | HPyV12 | YP_007684356 |
| Polyomaviridae | Vervet monkey polyomavirus | YP_007188590 |
| Polyomaviridae | Chimpanzee polyomavirus | CBX23445 |
| Polyomaviridae | HPyV9 | YP_004243706 |
| Polyomaviridae | Dolphin polyomavirus | AGR44742 |
| Polyomaviridae | Goose hemorrhagic polyomavirus | NP_849170 |
| Polyomaviridae | Crow polyomavirus | YP_529828 |
| Polyomaviridae | Butcherbird polyomavirus | YP_008873519 |
